# ﻿*Nicotianagandarela* (Solanaceae), a new species of ‘tobacco’ highly endangered from the Quadrilátero Ferrífero in Brazil

**DOI:** 10.3897/phytokeys.190.76111

**Published:** 2022-02-25

**Authors:** Mariana Augsten, Pablo Burkowski Meyer, Loreta B. Freitas, João A. N. Batista, João Renato Stehmann

**Affiliations:** 1 Departamento de Botânica, Instituto de Ciências Biológicas, Universidade Federal de Minas Gerais – UFMG, Av. Antônio Carlos, 6627, Pampulha, Belo Horizonte, CEP 31270–901, MG, Brazil Universidade Federal de Minas Gerais – UFMG Belo Horizonte Brazil; 2 Departamento de Genética, Universidade Federal do Rio Grande do Sul, 91501–970, Porto Alegre, RS, Brazil Universidade Federal do Rio Grande do Sul Porto Alegre Brazil

**Keywords:** Endemism, IUCN, Minas Gerais, mining, molecular phylogeny

## Abstract

*Nicotianagandarela* Augsten & Stehmann (Solanaceae), **sp. nov.**, a small ’tobacco’ known only from one locality at Serra do Gandarela, in the state of Minas Gerais, Brazil, is described and illustrated. It is morphologically characterized by its rosulate basal leaves, red corolla with a short tube not inflated at the apex, and the peculiar habitat, a shaded site under a rocky outcrop ledge along a forested stream. Phylogenetic analyses based on a combined dataset of nuclear (ITS) and plastid (*ndhF*, *trnLF*, and *trnSG*) DNA sequences revealed that the species belongs to the Nicotianasect.Alatae and is sister to the clade with the remaining species in the section. A key for the identification of Brazilian species of the section is given. The unusual habitat, the small population size, and the intense pressure of mining activities in the surroundings made the species assessed as Critically Endangered (CR), needing conservation efforts to avoid its extinction.

## ﻿Introduction

*Nicotiana* L. is a Solanaceae genus known mostly because of tobacco (*N.tabacum* L.), a crop cultivated worldwide, but its diversity goes further. It is the fifth-largest genus in the family, with 82 species (Knapp 2020). *Nicotiana* occurs in South America and Australia, with one species from Africa (Namibia). The Andean region is recognized as the center of diversity of the group in the Americas, as for many other clades in Solanaceae (Goodspeed 1954; Dupin et al. 2017). Molecular phylogenies are available for the genus (Aoki and Ito 2000; Chase et al. 2003; Clarkson et al. 2004; Clarkson et al. 2017), and sectional revision is provided based on phylogenetic analyses, indicating 13 sections (Knapp et al. 2004).

In Brazil, the known native species belong to the Nicotianasect.Alatae Goodsp. (hereafter Alatae). This section is morphologically characterized by herbaceous habit, rosulate and sessile leaves, usually viscid pubescent, few leaves on upper stems, and an abrupt dilatation at the throat of the corolla tube (Knapp et al. 2004). The section comprises two cytogenetic groups: one displays chromosome number 2n = 20 and includes two species, *N.longiflora* Cav. and *N.plumbaginifolia* Viv., distributed from Argentina to the USA; the other presents chromosome number 2n = 18 and includes *N.alata* Link & Otto, *N.bonariensis* Lehm., *N.forgetiana* Hemsl., *N.langsdorffii* Weinm., *N.mutabilis* Stehmann & Semir and *N.azambujae* L. B. Sm. & Downs, occurring in Brazil, Argentina, Uruguay, and Paraguay (Goodspeed 1954; Clarkson et al. 2004; Knapp et al. 2004). The internal phylogenetic relationships of the section are still not well resolved, with the two groups recovered as sisters (Clarkson et al. 2004) or with the 2n = 18 group as paraphyletic (Clarkson et al. 2017). According to the plastid or nuclear markers used for the reconstruction, section diversification is proposed between 6.2 Ma and 7.7 Ma, respectively (Clarkson et al. 2017).

*Nicotiana* can be considered a taxonomically well-studied genus for the Brazilian flora (Vignoli-Silva and Mentz 2005; Vignoli-Silva and Stehmann 2020), in which six native, *N.alata*, *N.bonariensis*, *N.langsdorffii*, *N.forgetiana*, *N.mutabilis*, and *N.azambujae*, and two naturalized species, *N.tabacum*, and *N.glauca* Graham, are recognized. Morphometric analyses based on the corolla size and shape of *N.forgetiana* throughout its geographical distribution showed that the species seems to include two distinct taxonomic groups, one not yet formally described (Teixeira et al. 2021). Among the Brazilian species, only *N.mutabilis* is considered endangered (Vulnerable) because of its small geographic distribution and just a few known populations (Stehmann et al. 2002; CNCFlora [http://cncflora.jbrj.gov.br/portal/pt-br/profile/Nicotiana%20mutabilis]). *Nicotianaazambujae* needs further investigation, as it is only known from the type collection.

Within Brazil, the Southern region holds the largest number of *Nicotiana* species, which inhabit ecotonal environments between the grasslands and the *Araucaria* forest (Mixed Ombrophilous Forest) in the Atlantic Forest domain (Teixeira et al. 1986; Overbeck et al. 2007). This is an important biogeographic area for several taxa that have their evolutionary history related to this vegetation mosaic (Iganci et al. 2011; Fregonezi et al. 2013). Species richness decreases towards the north, with *N.bonariensis* being the species with the northern-most distribution, reaching the grasslands at the high altitude regions of the Mantiqueira Range (Minas Gerais and Rio de Janeiro states) and the Espinhaço Range (Minas Gerais and Bahia states).

During floristic studies in the Quadrilátero Ferrífero, a mountainous area with large mineral reserves in Minas Gerais, with significant biological importance and high anthropic pressures (Drummond et al. 2005; Jacobi et al. 2011), samples of a small *Nicotiana* specimen with red flowers were collected. As the phenotype matched with none of the known *Nicotiana* species from Brazil, we assumed it could be a new species. In this work, we test this hypothesis using morphological and molecular data.

## ﻿Methods

### ﻿Taxonomy

Plant samples were collected during field expeditions in April 2018, and voucher specimens were deposited in the BHCB herbarium at the Universidade Federal de Minas Gerais, whereas flowers were preserved in Ethanol 50%. We took measures of vegetative parts from herbarium samples and of entire flowers from fixed material. Descriptive terminology was based on Radford et al. (1976). To compare the morphological characters with congeneric species and build the key, we revised the main taxonomic literature (Goodspeed 1954; Vignoli-Silva and Mentz 2005; Cocucci 2013; Vignoli-Silva and Stehmann 2020), including the protologues, as well as the materials from the following Brazilian herbaria: BHCB, HUEFS, ICN, MBM, OUPR, PAMG, RB, R, SP, SPF, and UEC. We also examined high-resolution images of the types available at Global Plants (https://plants.jstor.org).

### ﻿Sampling for phylogenetic analyses and molecular methods

We included DNA sequences from 56 *Nicotiana* species, mostly generated in previous studies (Chase et al. 2003; Clarkson et al. 2004) and obtained from GenBank (www.ncbi.nlm.nih.gov/Genbank). We selected species from all described sections (Knapp et al. 2004) and used two *Anthocercis* Labill. as outgroups. We generated DNA sequences for three different individuals of the new species, also for *N.mutabilis*, described in 2002 (Stehmann et al. 2002) and for which there were no DNA sequences available in the databases. For all Genbank accessions used in this work, see Suppl. material 1: Table S1. Fresh, young leaves were collected in the field and stored in silica gel. Total DNA was obtained using a modified version of the 2X-CTAB extraction method (Doyle and Doyle 1987) using 50 mg of tissue mass and washing with 70% ethanol. We used nucleotide sequences from the nuclear marker (ITS - rDNA internal transcribed spacers) and three plastid regions (*trnLF*, *trnSG*, and *ndhF*). The selection of markers was based on previous studies (Chase et al. 2003; Clarkson et al. 2004). Amplifications were performed using the same conditions described in previous works (Chase et al. 2003; Clarkson et al. 2004). Polymerase chain reaction (PCR) products were purified by precipitation with 20% PEG (2.5 M NaCl and 20% polyethylene glycol 8000) and sequenced on demand by a specialized company using the same primers employed for amplification. We obtained bidirectional sequence reads for all the DNA regions, and consensus sequences were obtained using Geneious Prime 2020.0.3 (https://www.geneious.com). Sequences were aligned using CLUSTALW (Larkin et al. 2007) followed by manual adjustments in MEGA 11 (Tamura et al. 2021). Sequences were concatenated with MESQUITE (Maddison and Maddison 2019), and we obtained the best substitution model per DNA region using MRMODELTEST2 (Nylander 2004).

### ﻿Phylogenetic analyses

We analyzed the data using Bayesian inference (BI) and Maximum Likelihood (ML). Initially, nuclear and plastid DNA were separately analyzed and posteriorly combined. We performed BI analyses using MRBAYES 3.2.7a (Ronquist et al. 2012) as implemented in the Cyberinfrastructure for Phylogenetic Research (CIPRES) Portal 2.0 (Miller et al. 2010), treating each DNA region as a separate partition. The “unlink” command was used to estimate model parameters separately for each partition. Each analysis consisted of two independent runs with four chains for 7,500,000 generations, sampling one tree every 1,000 generations and a temperature parameter of 0.2. Convergence between the runs was evaluated using the average standard deviation of split frequencies (< 0.01) and the Potential Scale Reduction Factor – PRSF (= 1.0) and was achieved after 715,000 generations. After discarding the first 2,500 trees (33%) as the burn-in, the remaining trees were used to assess topology and posterior probabilities (PP) in a 50% majority-rule consensus. We considered groups with PP > 0.95 as strongly supported, groups with PP ranging from 0.90–0.95 as moderately supported, and groups with PP < 0.90 as weakly supported. Maximum likelihood (ML) analysis of the concatenated dataset was performed using the online version of RAxML-HPC BlackBox (v.8.2.12) (Stamatakis 2014) through the CIPRES Science Gateway Portal (Miller et al. 2010). Bootstrap values were calculated based on 1000 replicates.

### ﻿Conservation status

The conservation status was assessed based on the International Union for Conservation of Nature Criteria (IUCN 2019), considering the main threats to the Quadrilátero Ferrífero vegetation (Sonter et al. 2014).

### ﻿Scanning electron microscopy analysis

Seeds were affixed in aluminum stubs using double-sided carbon tape, metalized with 10 nm gold-palladium (Robards 1978). The observations were performed under a JEOL JSM– 6360LV scanning electron microscope, with 5 kV (Jeol Ltd., Tokyo, Japan).

## ﻿Results

### ﻿Alignment characterization

The concatenated matrix consisted of 3,483 aligned characters, of which 317 (9.1%) were potentially informative. The ITS region had, proportionately, the highest number of phylogenetically-informative characters (20%). General features of the datasets and a summary of the models implemented for each partition are presented in Table 1.

**Table 1.** General features of the markers used in the phylogenetic analyses and models implemented for each.

Regions	Terminals	Characters	Variable uninformative characters	Informative characters (%)	Model
ITS	59	683	89	135 (20%)	GTR+I+G
*ndhF*	60	1056	49	71 (6.7%)	GTR+G
*trnLF*	60	991	41	58 (5.8%)	GTR+G
*trnSG*	60	855	38	62 (7.2%)	GTR+G
All plastid regions	60	2799	125	182 (6.5%)	–
All regions	60	3483	214	317 (9.1%)	–

### ﻿Phylogenetic relationships

In the Bayesian phylogenetic tree based on ITS, *N.gandarela* appears within the section Alatae, the Alatae clade is moderately supported (PP = 0.91), and *N.gandarela* is sister of the 2n = 20 clade (*N.longiflora* and *N.plumbaginifolia*), being weakly supported (PP = 0.76) (Suppl. material 2: Fig. S1). The concatenated plastid regions recovered a Bayesian phylogenetic tree with *N.gandarela* as the sister species of section Noctiflorae, moderately supported (PP = 0.91), and Alatae appears as sister of section Repandae, weakly supported (PP = 0.84) (Suppl. material 3: Fig. S2).

Due to the combined dataset produced higher-supported trees in both ML and BI methods than using nuclear and plastid regions independently, we described the results based on this supermatrix only. As the topology was similar in ML and BI trees, we detailed the species relationships from BI results only. The three individuals of *N.gandarela* formed a well-supported clade in all obtained trees.

**Figure 1.**
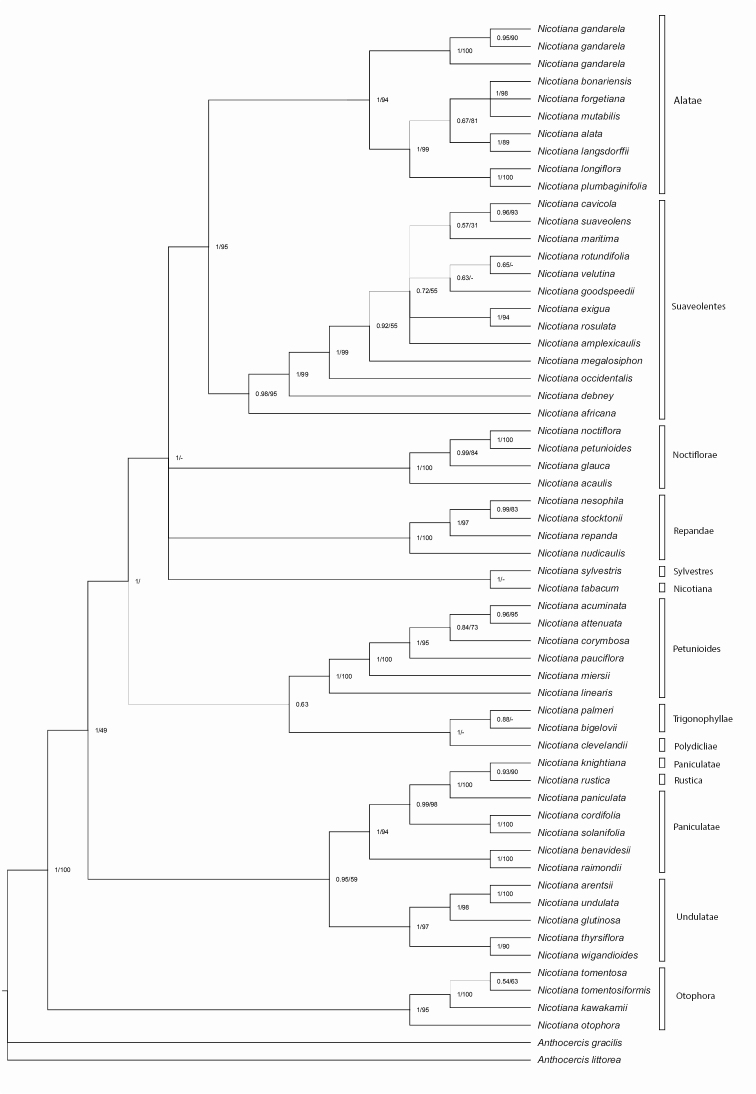
Bayesian 50% majority-rule consensus tree of 56 species of *Nicotiana* L. of the combined nuclear (ITS) and plastid (*ndhF*, *trnLF*, and *trnSG*) datasets. Numbers next to the nodes represent the posterior probabilities (PP) and bootstrap values. Section names (according to Knapp et al. 2004) are indicated on the right. Width of lines defined by PP values.

The *Nicotiana* species analyzed formed a strongly supported clade (PP = 1.00), and several subclades corresponded to the accepted sections for the genus (Fig. 1). The analysis confirmed the monophyly of the Alatae (PP = 0.99), which is strongly supported as the sister of the Nicotianasect.Suaveolens Goodsp. (PP = 1.00). The two newly sequenced species, *N.gandarela* and *N.mutabilis*, were resolved as members of the Alatae. *Nicotianagandarela* was recovered with high support (PP = 0.99) as sister to the clade with the remaining species in the Alatae, whereas *N.mutabilis* was resolved as closely related to *N.forgetiana* and *N.bonariensis* (PP = 1.00). Similar to previous analyses, low support was observed for the 2n = 18 group and for the relationship of *N.bonariensis* to *N.langsdorffii* (PP = 0.66).

## ﻿Taxonomic treatment

### ﻿ Nicotiana gandarela

Taxon classificationPlantaeSolanalesSolanaceae

Augsten & Stehmann sp. nov.

86855F52-FEEE-5486-9F57-EE7A14E6B00E

urn:lsid:ipni.org:names:77260722-1

Fig. 2

#### Type.

Brazil. Minas Gerais. Santa Bárbara, André do Mato Dentro, 19°59'43"S, 43°38'39"W, 17 Apr 2018 (fl, fr), *M. Augsten & J.R. Stehmann* 1078 (holotype: BHCB acc.#190958 [BHCB190958!]; isotypes: ICN, RB, to be distributed).

#### Diagnosis.

*Nicotianagandarela* differs from all other species of the Nicotianasect.Alatae by its short corolla tube (< 15 mm), vivid red corolla limb, and unusual shaded, cave-mouth habitat.

#### Description.

Annual herb to 0.5 m high, not branched, pubescent with multicellular, glandular trichomes. Leaves simple, rosulate, crowded, 6–21 cm long, (1.7–) 3.5–5.0 cm wide, persistent, spatulate, slightly discolorous, the blade hirsute throughout with simple, predominantly glandular hairs, long-attenuate at the base, margin sinuous, ciliate, midribs, and secondary veins visible at both surfaces, rounded or obtuse, sometimes acute, at the apex. Inflorescence scapose, paniculate, apical or lateral, composed of monochasial cymes; bracts lanceolate, 4.3 mm long, 1.0 mm wide, viscid-hirsute; pedicels 5.8–7.7 mm long. Calyx hirsute-glandular, deeply lobed, the tube 2.5 mm long, lobes 3–5 mm long, lanceolate, unequal, the apex acute. Corolla aestivation conduplicate, with folded basal petals covering the other three; tube 1.2–1.5 cm long, 3.0–3.4 mm diam., infundibuliform, not inflated at the apex, magenta, trichomes rare, opening ca. 3.4 mm; limb zygomorphic, red, cleft into widely-depressed ovate to very widely ovate lobes, patent or slightly reflexed. Stamens 5, didynamous, adnate ca. 5 mm from the base of the corolla tube, four longer, ca. 12.5 mm long, one shorter, ca. 11.4 mm long, all filaments glabrous; anthers 0.9–1.2 mm long, white, ellipsoid, pollen whitish; nectariferous disk present. Ovary slightly conical, glabrous, style ca. 9.4 mm long, stigma bilobed, green. Capsule 2-valved, included in the calyx, 6.0–7.5 mm long, 4.4–5.5 mm in diameter, ovoid, valves with incised apex. Seeds about 0.7–0.8 mm long, 0.6–6.5 mm wide, subglobose to obovoid, testa foveolate, anticlinal walls sinuous. Chromosome number unknown.

#### Etymology.

The specific epithet “gandarela” is a noun in apposition and refers to Serra do Gandarela, the mountain range complex where this species is found.

**Figure 2.**
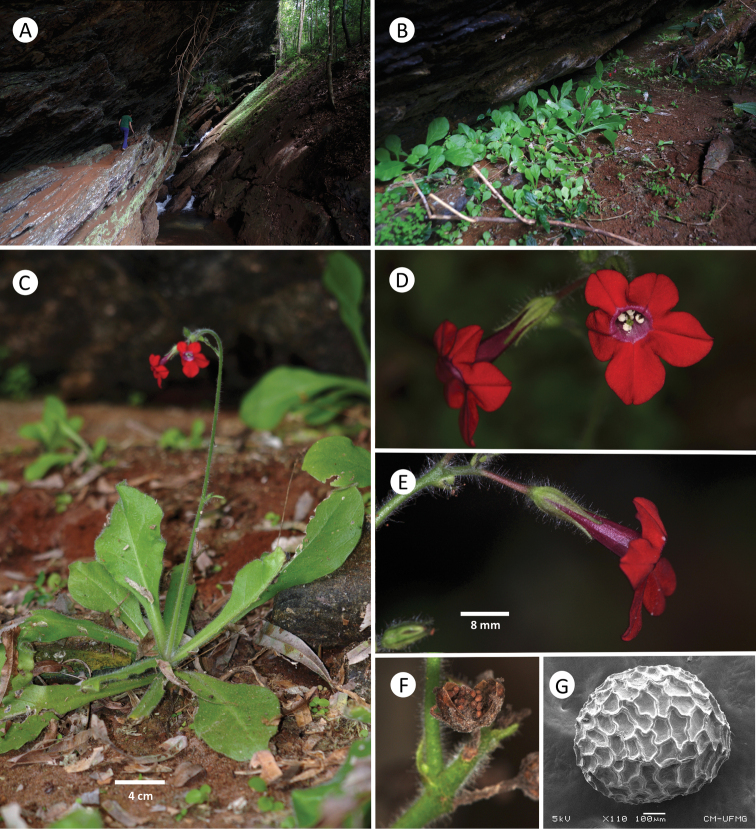
*Nicotianagandarela* Augsten & Stehmann **A** peculiar habitat of the species, the shaded sites in the base of the shaded ledge **B** seedlings growing in the site **C** habit highlighting the rosulate leaves and the scapose inflorescence **D, E** flowers in lateral and frontal view **F** 2-valvate capsule with many seeds. **G** seed with sinuous anticlinal walls (Scanning Electron Microscopy) **D–G** were obtained from plants of the type population (Augsten and Stehmann 1078, BHCB). Photos by JR Stehmann.

#### Distribution and habitat.

**Figure 3.**
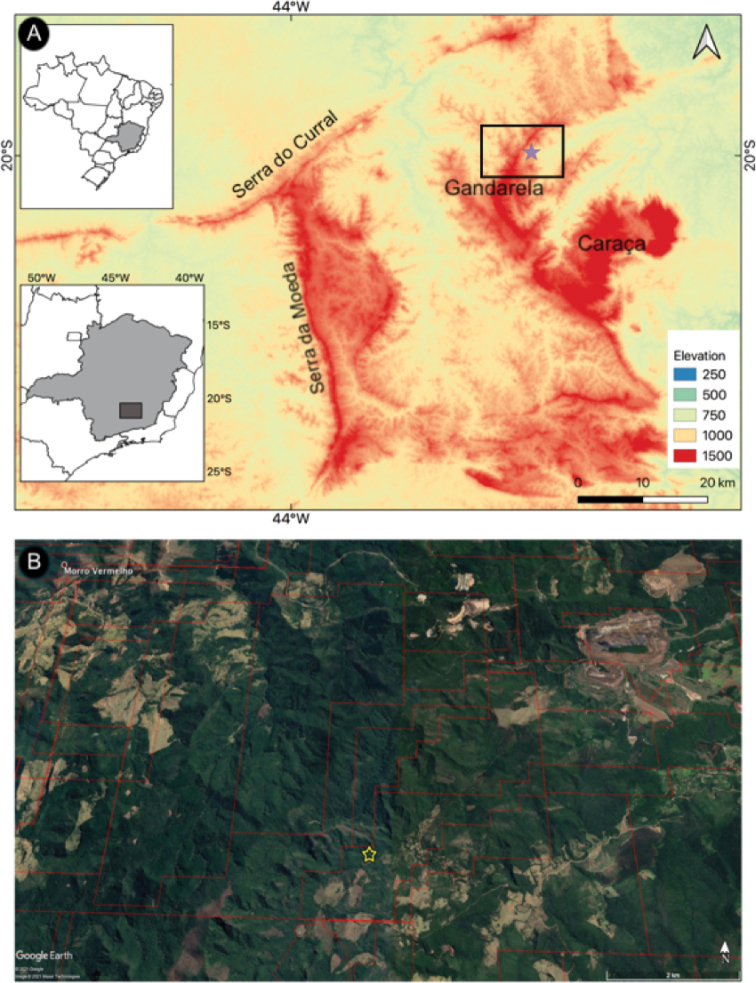
**A** main mountains of the Quadrilátero Ferrífero in Minas Gerais. Star indicates the *Nicotianagandarela* Augsten & Stehmann occurrence site **B** Google Earth image showing the regional landscape associated with the *N.gandarela* site, the forest matrix impacted by a small village, and open mining. The red lines indicate mining concessions (Instituto Prístino: Atlas Digital Geoambiental [https://institutopristino.org.br/atlas/]).

The only known population of *Nicotianagandarela* is at the Serra da Gandarela Mountain range, located in the northeast portion of the Quadrilátero Ferrífero in the Minas Gerais Brazilian State (Fig. 3A). The species occurs in a shaded site placed under a rocky outcrop ledge that extends for about 350 m along a stream and surrounded by a forest matrix, making this area a permanent humid environment. We recognized three subpopulations, each up to 150 individuals, including many seedlings (Fig. 2B). This environment resembles an open cave, and the individuals grow in bare powdery red soil originating from rock decomposition, usually with no other species co-occurring. The habitat is unique, and no similar microhabitat exists in this geologically diverse and complex area (Instituto Prístino: Atlas Digital Geoambiental: Quadrilátero Ferrífero: Geodiversidade [https://institutopristino.org.br/atlas/quadrilatero-ferrifero/]) Fig. 2A–B.

#### Phenology.

*Nicotianagandarela* has been collected with flowers and fruits in December and April.

#### Conservation status.

Although the single site for *N.gandarela* would normally suggest the species was Data Deficient (DD), the precarious nature of the region in which it grows leads us to assess it more formally. The species was preliminarily assessed as Critically Endangered – CR (B1, B2a, biii, D), mainly due to its geographic range, as the extent of occurrence (EOO) and the area of occupancy (AOO) are smaller than 100 km^2^ and 10 km^2^, respectively. The only populations of *N.gandarela* are located on private property and not inserted in any integral conservation unit. In addition, the Quadrilátero Ferrifero suffers from constant habitat loss (Salles et al. 2018) as well as strong pressures from the mining sector (Sonter et al. 2014) with concessions required to explore iron and gold (Instituto Prístino: Atlas Digital Geoambiental 2021 [https://institutopristino.org.br/atlas/quadrilatero-ferrifero/]) (Fig. 3B).

#### Additional specimen examined.

Brazil. **Minas Gerais**: Santa Bárbara. André do Mato Dentro, trilha para a cachoeira, 31 Dec 2017 (fl.,fr.), *D.M.G. Oliveira*, *P.L.Viana & N.O.Mota 359* (BHCB).

### ﻿Identification key to the Brazilian species of Nicotianasect.Alatae

**Table:** 

1	Calyx length equal to the corolla tube	***N.azambujae* L.B. Sm. & Downs**
–	Calyx length shorter than the corolla tube	**2**
2	Corolla yellow-greenish or light green, actinomorphic, limb almost entire (shortly lobed), pollen blue	***N.langsdorffii* Weinm.**
–	Corolla white, pink, magenta, purple or red, zygomorphic, limb clearly lobed; pollen green, white or yellow	**3**
3	Corolla tube > 30 mm long, limb usually white, filaments adnate to the upper portion of the corolla	**4**
–	Corolla tube usually < 30 mm long, limb rarely white, filaments adnate to the lower portion of the corolla tube	**5**
4	Leaves decurrent, limb strongly zygomorphic, stamens inserted in two levels (4 higher and 1 lower) at the corolla tube, filaments all geniculate	***N.alata* Link & Otto**
–	Leaves auriculate, not decurrent, limb weakly zygomorphic, stamens inserted in three levels (2 higher, 2 middle, and 1 lower) at the corolla tube, filaments not geniculate	***N.longiflora* Cav.**
5	Corolla changing from white to pink or magenta during the anthesis	***N.mutabilis* Stehmann & Semir**
–	Corolla keeping the same color during the anthesis	**6**
6	Leaves evenly distributed and decurrent along the stem; calyx usually > 9 mm; corolla tube usually > 20 mm long, magenta or purple-red	***N.forgetiana* Hemsl.**
–	Leaves usually rosulate, not decurrent on the stem; calyx usually < 9 mm long; corolla tube < 20 mm long, limb red or white	**7**
7	Corolla tubular, distally ventricose; limb white	***N.bonariensis* Lehm.**
–	Corolla tube gradually enlarged to the apex, not ventricose, limb red	***N.gandarela* Augsten & Stehmann**

## ﻿Discussion

Overall, the recovered phylogenetic relationships were similar to those previously obtained (Chase et al. 2003; Clarkson et al. 2004; Knapp et al. 2004; Clarkson et al. 2017). Phylogenetic trees generated showed that the samples collected in the Serra da Gandarela in Minas Gerais are genetically distinct from all known species of *Nicotiana* used in our analyses. *Nicotianagandarela* was recovered as the sister of all other species belonging to the Alatae (both of the groups with distinct cytotypes, 2n = 18 and 2n = 20; Clarkson et al. 2004; Knapp et al. 2004). The internal nodes of the group 2n = 18 showed low support, and the lack of chromosome accounts to the new species does not permit any inferences about the phylogenetic relationships within the section.

The new species is an annual plant, with rosulate leaves, long-attenuate to the base, sessile, viscid-pubescent, and a zygomorphic corolla, all morphological traits associated with the species of the Alatae (Knapp et al. 2004). The decurrent leaf bases on the stem and the abrupt dilatation at the corolla throat, commonly found in the other species of the section (Goodspeed 1954), are lacking in the new species. The habit resembles that of *N.bonariensis* because of its rosulate leaves and scapose inflorescence. However, *N.gandarela* differs from *N.bonariensis* in its ombrophilous and extremely narrowly distributed habitat, its diurnal anthesis with red corollas with tube < 15 mm long, rather than being being heliophilous, widespread, with nocturnal anthesis, and white corollas with a tube usually > 15 mm long.

Corolla color is variable in the Alatae, including white, yellow, pink, magenta, and purple-red. Such variation reflects an evolutionary history of radiation to distinct pollinator agents (Ippolito et al. 2004; Kaczorowski et al. 2005; Knapp 2010). Red flowers are usually associated with bird pollination (Faegri and van der Pijl 1979), and hummingbirds were already reported pollinating *N.forgetiana*, a pink to purple-red flowered species distributed in southern Brazil (Ippolito et al. 2004). Empirical data on the effective pollinator of *N.gandarela* are still necessary.

The Quadrilátero Ferrífero in Minas Gerais is located in the southern Espinhaço Range, a mountainous chain where the Cerrado (tropical Savannah) and Brazilian Atlantic Forest, two hotspots of biodiversity, connect (Mittermeier et al. 2004). The vegetation is a mosaic of topologies and vegetation, including grasslands, savannah, and forests (Spósito and Stehmann 2006), whose distributions are influenced by an altitudinal gradient, ranging from about 700 m to 2,080 m (Borges et al. 2011). In the region, besides the new species, a further two species of the Alatae are found, *N.bonariensis* and *N.langsdorffii*. The first usually inhabits the grasslands at higher altitudes known as Campos Rupestres (Silveira et al. 2016), while the second is generally associated with disturbed areas. Both grow mainly in open and sunny sites, as with most species of the Alatae, and the peculiar, shaded habitat of *N.gandarela* seems to be unique in the section. The microhabitat of the new species roughly resembles that of *Petuniaexserta* Stehmann, an endemic species from Rio Grande do Sul, in Southern Brazil, but where the geology and vegetational matrix are totally different (Lorenz-Lemke et al. 2006; Stehmann et al. 2009). In both cases, few or no species share the ground where these two species grow, meaning that they are fragile, with low, competitive capacity, but are presumably adapted to survive in these empty niches. Penumbral plant communities have also been described for the Campos Rupestres in Minas Gerais, where several species were reported as growing in small caves (Alves and Kolbek 1993), but until now, no Solanaceae species have been registered in these areas.

The new species deserves special conservation attention because it inhabits a small and rare habitat, if not unique, in the Quadrilátero Ferrífero, a region that is suffering intensive habitat loss (Salles et al. 2018) and has been the scenario of recurrent environmental disasters (Carmo et al. 2017; Rotta et al. 2019). Floristic inventories looking for new populations should be carried out in the surrounding forested areas, including the Serra do Gandarela National Park, following the drainage lines in the valleys. Also, we suggest the engagement of local people in trying to find other populations because of the impossibility of looking for similar areas since they are hidden by forest in satellite images. In the end, although we emphasize that the species should be primarily preserved in its natural habitat, ex-situ conservation measures might also be necessary (CNCFlora 2016) in order to prevent its extinction.

## Supplementary Material

XML Treatment for Nicotiana gandarela

## Supplementary materials

Supplementary material 1 Table S1 Data type GenBank accesions Explanation note Genbank accessions. ITS sequences from Chase et al. 2003, Plastidial sequences (*trnL-F*, *trnS-G* and *ndhF*) from Clarkson et al. 2004. Sequences from this work marked with an * (*Nicotianagandarela* and *Nicotianamutabilis*). https://binary.pensoft.net/file/650762This dataset is made available under the Open Database License (http://opendatacommons.org/licenses/odbl/1.0/). The Open Database License (ODbL) is a license agreement intended to allow users to freely share, modify, and use this Dataset while maintaining this same freedom for others, provided that the original source and author(s) are credited.Mariana Augsten, Pablo Burkowski Meyer, Loreta B. Freitas, João A. N. Batista, João Renato Stehmann

Supplementary material 2 Figure S1 Data type Phylogenetic Explanation note Bayesian 50% majority-rule consensus tree of 56 species of *Nicotiana* L. of the nuclear (ITS) dataset. Numbers next to the nodes represent the posterior probabilities (PP). https://binary.pensoft.net/file/650763This dataset is made available under the Open Database License (http://opendatacommons.org/licenses/odbl/1.0/). The Open Database License (ODbL) is a license agreement intended to allow users to freely share, modify, and use this Dataset while maintaining this same freedom for others, provided that the original source and author(s) are credited.Mariana Augsten, Pablo Burkowski Meyer, Loreta B. Freitas, João A. N. Batista, João Renato Stehmann

Supplementary material 3 Figure S2 Data type Phylogenetic Explanation note Bayesian 50% majority-rule consensus tree of 56 species of *Nicotiana* L. of the plastid (*trnL-F*, *trnS-G* and *ndhF*) datasets. Numbers next to the nodes represent the posterior probabilities (PP). https://binary.pensoft.net/file/650764This dataset is made available under the Open Database License (http://opendatacommons.org/licenses/odbl/1.0/). The Open Database License (ODbL) is a license agreement intended to allow users to freely share, modify, and use this Dataset while maintaining this same freedom for others, provided that the original source and author(s) are credited.Mariana Augsten, Pablo Burkowski Meyer, Loreta B. Freitas, João A. N. Batista, João Renato Stehmann
