# Supplementary figures and images for: ﻿Nicotianagandarela (Solanaceae), a new species of ‘tobacco’ highly endangered from the Quadrilátero Ferrífero in Brazil
Source: PhytoKeys. 2022 Feb 25;190:113–29. doi: 10.3897/phytokeys.190.76111 (PMC8897371; doi:10.3897/phytokeys.190.76111)

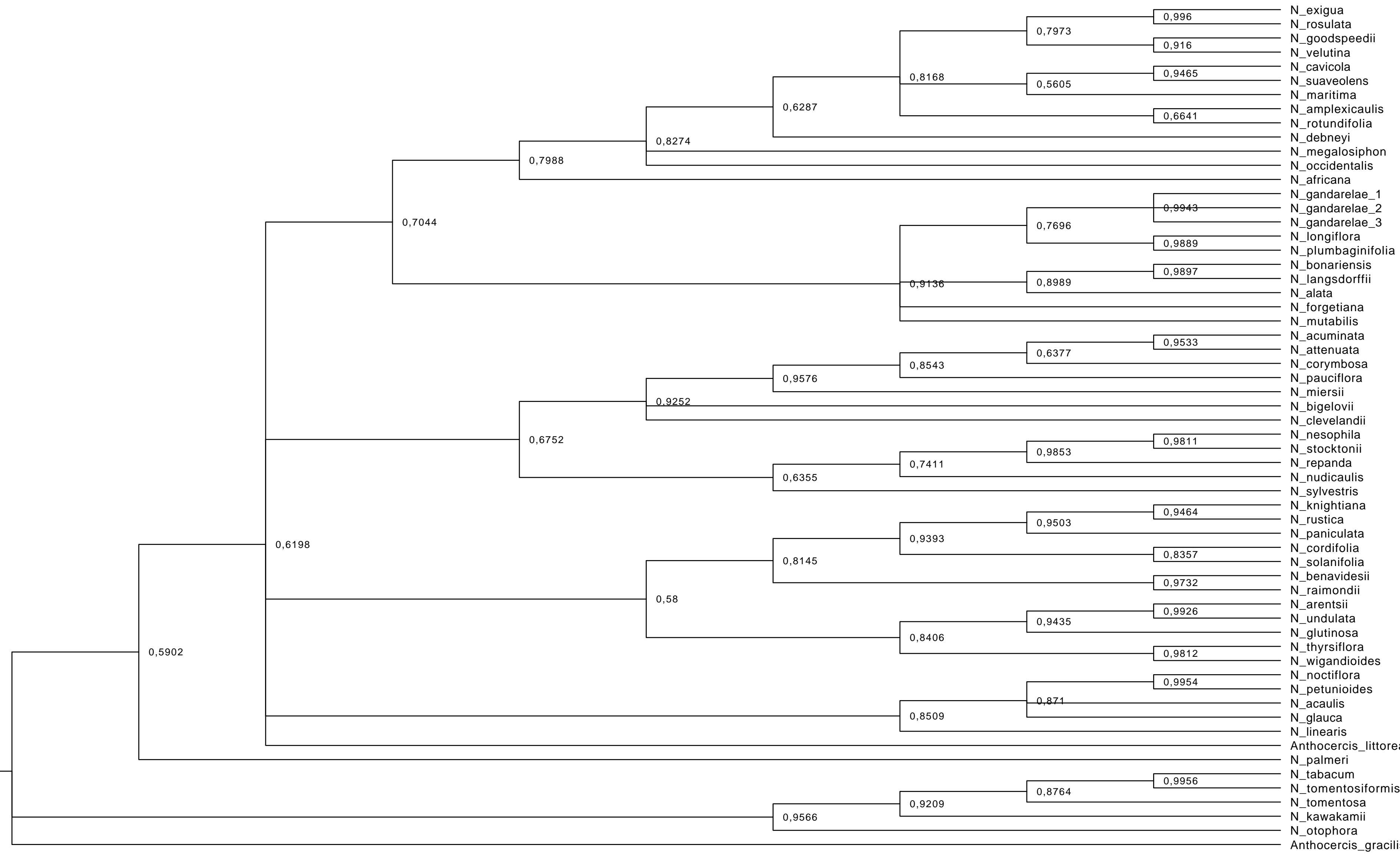

Supplement: Supplementary material 2 — Figure S1 [file phytokeys-190-113-s002.pdf]

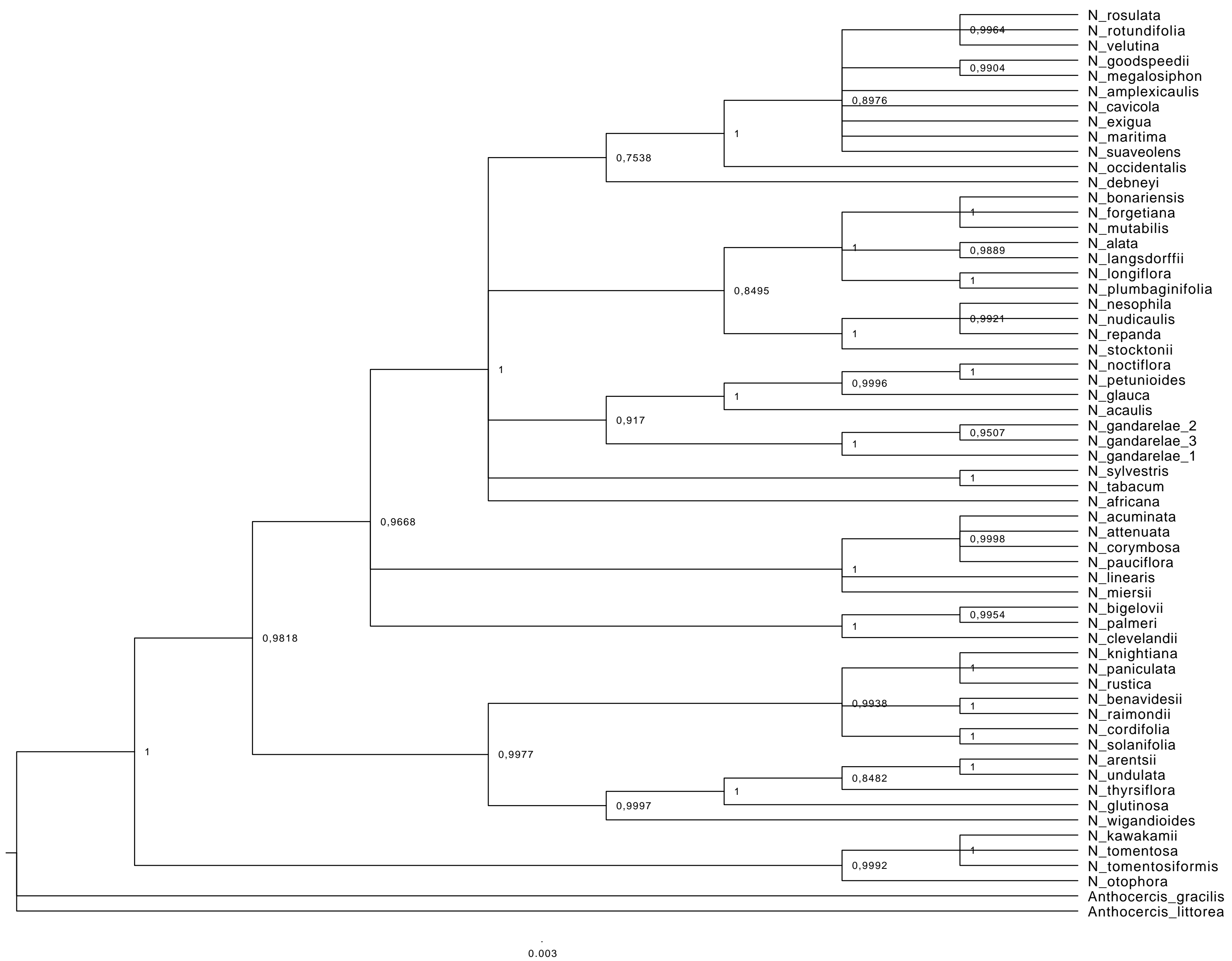

Supplement: Supplementary material 3 — Figure S2 [file phytokeys-190-113-s003.pdf]
